# Cutaneous Coinfection of Cytomegalovirus and *Mycobacterium chelonae* Accelerated by Immunosuppression

**DOI:** 10.1155/2021/8819560

**Published:** 2021-01-29

**Authors:** Yutaka Tsutsumi, Kentaro Odani, Yasuhito Kaneko, Hideo Hashizume, Mitsuhiro Tachibana

**Affiliations:** ^1^Diagnostic Pathology Clinic, Pathos Tsutsumi, Nagoya, Aichi, Japan; ^2^Department of Diagnostic Pathology, Shimada Municipal Hospital, Shimada, Shizuoka, Japan; ^3^Department of General Medicine, Shimada Municipal Hospital, Shimada, Shizuoka, Japan; ^4^Department of Dermatology, Shimada Municipal Hospital, Shimada, Shizuoka, Japan

## Abstract

A mildly diabetic 58-year-old male had traumatic ulceration on the left popliteal fossa, and the lesion progressed to a painful 6 cm deep ulcer. After surgical debridement and skin grafting, ulceration recurred. Pyoderma gangrenosum was clinically diagnosed after the first biopsy, indicating a noninfective ulcer. Immunosuppressive therapy (prednisolone and cyclosporine A) induced complete epithelialization in three months. Four months later, subcutaneous nonulcerated nodules appeared on the anterior area of the left lower leg. Subcutaneous induration progressed and ulceration recurred, so that immunosuppressive therapy continued for one year. Cytomegalovirus (CMV) viremia was detected, and the second biopsy demonstrated CMV inclusions of endothelial and perivascular cells in fibrosing septolobular panniculitis. Cyclosporine A was cancelled, prednisolone was tapered, and ganciclovir started. Viremia soon disappeared, but the lesion progressed to large induration with multiple ulcers measuring up to 3 cm. The third biopsy disclosed infection of Gram-positive mycobacteria, accompanying fat droplet-centered suppurative granulomas without CMV infection. Microbial culture identified *Mycobacterium chelonae*. Clarithromycin with thermotherapy was effective. A review of the second biopsy confirmed coinfection of CMV and Gram-positive mycobacteria. Immunostaining using a panel of anti-bacterial antibodies visualized the mycobacteria in the lesion. Positive findings were obtained with antibodies to Bacillus Calmette-Guérin, *Bacillus cereus*, MPT64 (*Mycobacterium tuberculosis*-specific 24 kDa secretory antigen), LAM (*Mycobacterium tuberculosis*-related lipoarabinomannan), and PAB (*Propionibacterium acnes*-specific lipoteichoic acid).

## 1. Introduction

Immunosuppressive conditions may provoke opportunistic skin infection of a variety of bacteria, fungi, and viruses [1, 2]. The present article describes a case of pyoderma gangrenosum on the left popliteal fossa, followed by immunosuppressive therapy-induced opportunistic dual skin infection of cytomegalovirus (CMV) and *Mycobacterium chelonae* on the left lower leg. Histopathological and immunohistochemical features of this rare combination of pathogens are detailed.

## 2. Case Report

**Figure 1:**
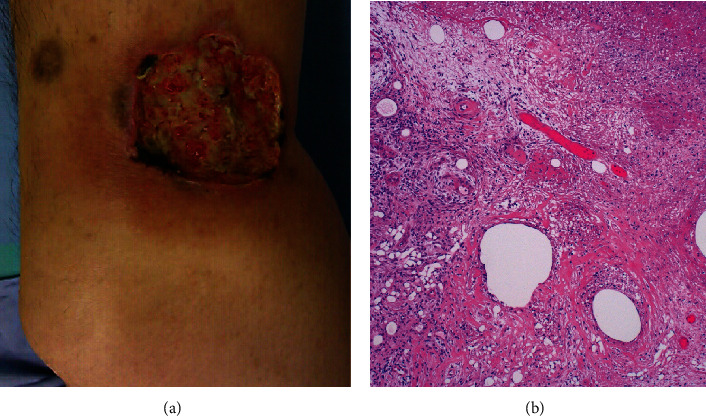
Pyoderma gangrenosum ((a) gross appearance of left lower thigh, (b) H&E). A demarcated deep ulcer, 4 cm in size, is observed. Fibrinous exudation is seen at the ulcer base, and erythematous change is associated around the ulcer (a). Microscopic features of the first biopsy specimen are nonspecific. Ulcer base is composed of fibrinous exudation and granulation tissue (b). Neither granulomatous reactions nor infectious agents are identified.

A 58-year-old Japanese man slipped and fell on the road to have his left knee contused, resulting in a 1.5 cm sized shallow ulceration. The patient had suffered from hypertension and mild type 2 diabetes mellitus for two years, but no history of autoimmune disorders. Serum antibodies against insulin and glutamic acid decarboxylase were negative, excluding the possibility of type 1 diabetes mellitus. He had smoked 15 cigarettes per day for more than 40 years. Five months later, a painful skin ulcer, 6 cm in size, occurred on the inner side of the left popliteal fossa (at the same site of contusion). No pathogens were identified in the biopsy specimen, and microbial culture was negative. Surgical debridement and full-thickness grafting from the abdominal skin were performed by a plastic surgeon, but half of the skin flap was eventually impaired, leaving a 3 cm sized deep ulcer with severe pain. The patient was then consulted to dermatologists. Clinical findings, as well as the first biopsy features displaying nonspecific and noninfective ulcer, were consistent with pyoderma gangrenosum (Figure 1). The serum level of granulocyte colony-stimulating factor, a biomarker of pyoderma gangrenosum [3], was elevated to 48.1 pg/mL (reference value < 30 pg/mL). Administration of prednisolone (PSL) (20 mg/day) and cyclosporine A (CyA: 100 mg/day) started. In order to control exacerbation of the skin lesion, medication of PSL plus CyA was maintained, while PSL was gradually tapered to 10 mg/day. Complete epithelialization was achieved three months later.

**Figure 2:**
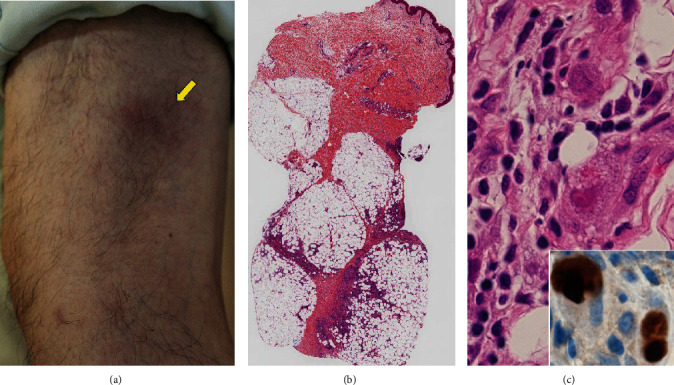
A subcutaneous nodule without ulceration ((a) gross appearance; (b) H&E, low-powered view; (c) H&E, high-powered view of the subcutaneous tissue, inset: immunostaining for CMV antigens). Subcutaneous nodularity with mild reddening is observed on the lower thigh near the original ulcer by pyoderma gangrenosum (arrow, (a)). A low-powered view of the second biopsy specimen demonstrates subcutaneous septolobular panniculitis (b). Endothelial cells and perivascular stromal cells with enlarged nuclei reveal intranuclear and cytoplasmic inclusions (c). Immunostaining for CMV antigens is clearly positive (inset).

**Figure 3:**
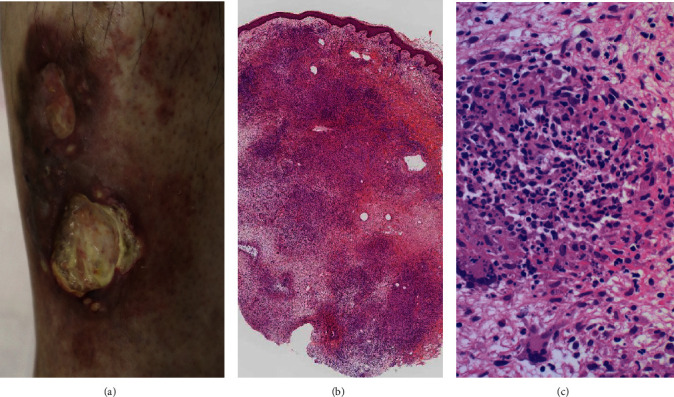
A large nodular and indurated skin lesion with multiple ulcers ((a) gross appearance, (b) H&E, low-powered view, and (c) H&E, high-powered view of the dermis). Multiple ulcers, 2–3 cm in size, are formed in the irregular-shaped reddish induration (a). The third biopsy specimen reveals diffuse inflammatory infiltration in the dermis through subcutaneous tissue (b). Suppurative granuloma (abscess surrounded by epithelioid granuloma) is scattered in the lesion (c). No CMV inclusions are observed any longer.

**Figure 4:**
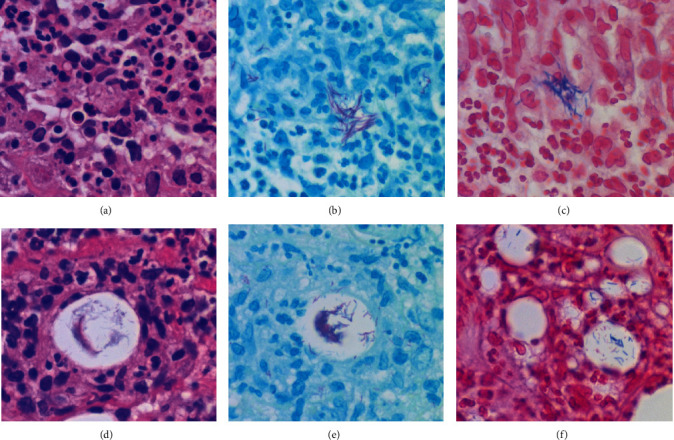
Gram-positive acid-fast bacilli in the second (a–c) and third (d–f) biopsy specimens ((a, d) H&E, (b, e) Ziehl-Neelsen stain, and (c, f) Gram stain). Gram-positive acid-fast bacilli are multifocally clustered in microabscess (a–c) and also in fat droplets located in the center of suppurative granuloma (d–f). The bacteria are visible in the fat droplets even in H&E preparations (d). Such patterns of bacterial colonization are seen in both the second and third biopsy specimens.

Four months later, subcutaneous nonulcerated, painful nodules appeared on the anterior area of the left lower leg, 20 cm distal from the primary ulcer caused by pyoderma gangrenosum. Ulceration recurred, and subcutaneous induration soon progressed toward both the distal and proximal directions. Immunosuppressive therapy restarted, but the painful ulcer persisted for one year. At this point of time, CMV viremia was identified, and the second skin biopsy from one of the subcutaneous nodules identified CMV inclusions in the subcutaneous tissue with fibrosing septolobular panniculitis. The CMV antigens were immunohistochemically visualized in the viral inclusions with 1 : 200 diluted cocktail monoclonal antibodies, CCH2+DDG9, available from Agilent Technologies (Figure 2). Both endothelial cells and perivascular stromal cells were infected. The dermis was free of inflammation. CyA was cancelled, PSL was reduced to 5 mg/day, and instead, ganciclovir (900 mg/day) was administered. Viremia soon disappeared, but the lower leg lesion progressed to form multiple ulcers. The third skin biopsy disclosed that infection involved both the dermis and the subcutaneous fat tissue. Suppurative granulomas (small abscesses surrounded by epithelioid granulomas) were dispersed (Figure 3). Gram-positive acid-fast bacilli were identified in microabscesses and also in fat droplets surrounded by suppurative granulomas (Figure 4). Caseous necrosis was focally associated. Grocott stain gave negative results. CMV infection was no longer identified. The second skin biopsy was retrospectively reevaluated, and Gram-positive acid-fast bacilli were identified mainly in microabscesses and in fat droplets surrounded by suppurative granulomas, multifocally distributed in the inflamed subcutaneous tissue. The final diagnosis of the second biopsy was thus subcutaneous coinfection of CMV and nontuberculous mycobacteria.

**Figure 5:**
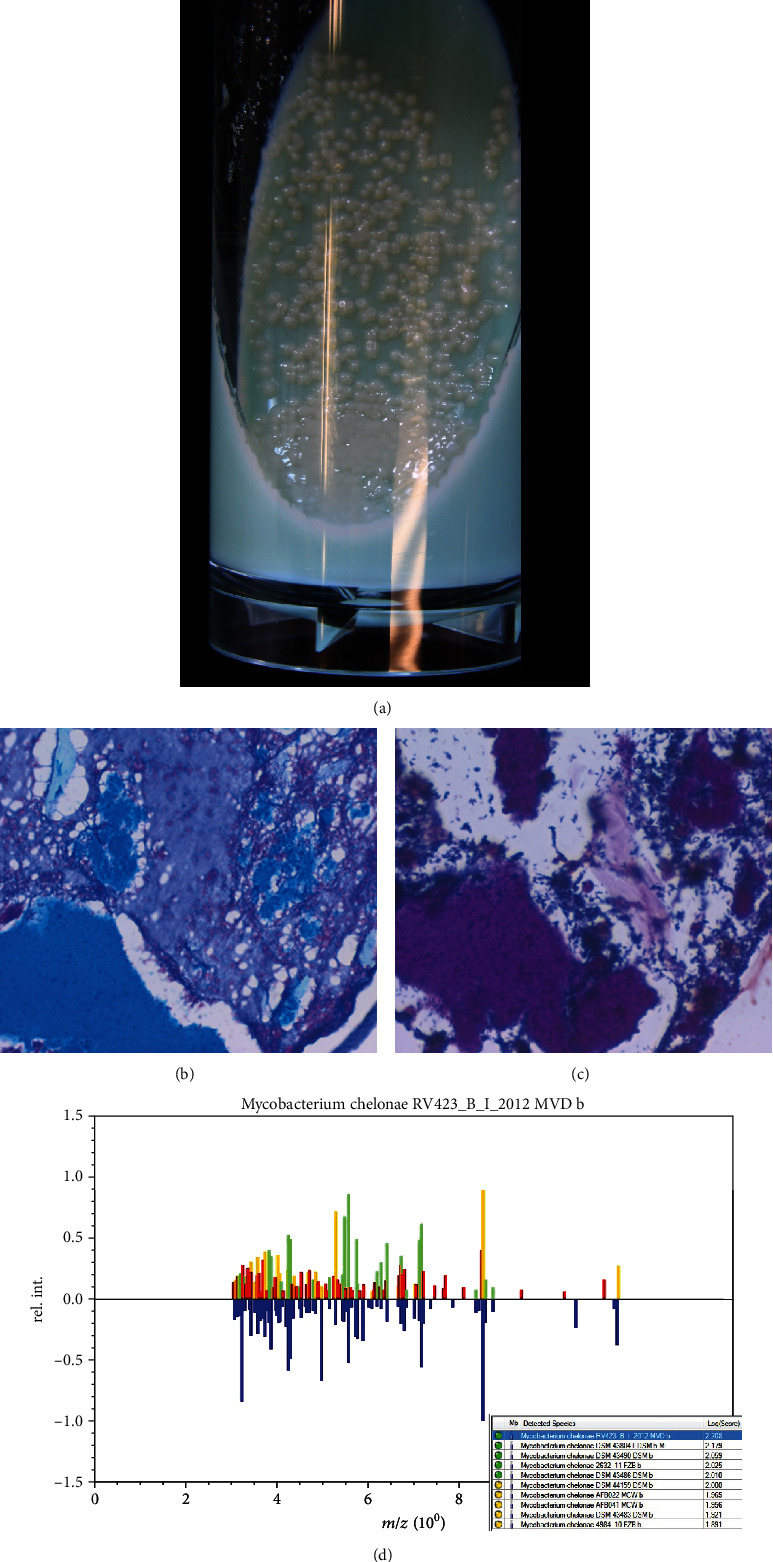
Colonies of *Mycobacterium chelonae* on 2% Ogawa medium ((a) gross appearance, (b) Ziehl-Neelsen stain, (c) Gram stain, and (d) MALDI Biotyper analysis). Smooth-surfaced white (nonchromogenic) colonies are formed seven days after inoculation. The formalin-fixed, paraffin-embedded colonies display acid-fastness and Gram reactivity of the rods. By the MALDI biotyping analysis, the spectrum pattern indicates *M. chelonae* with the log score value of 2.208.

Microbial culture on 2% Ogawa medium at 37°C identified rapidly growing nontuberculous mycobacteria. The bacteria were acid-fast and Gram-positive. *Mycobacterium chelonae* was identified by analyzing with Matrix-Assisted Laser Desorption/Ionization Time-of-Flight Mass Spectrometry (MALDI-TOF MS) by using the MALDI Biotyper (Bruker Japan Daltonics division, Yokohama) (Figure 5) [4]. The log score value for *M. chelonae* was more than 2.0 (2.208).

Clarithromycin administration (800 mg/day), combined with thermotherapy using disposal pocket body warmers for one hour twice a day, resulted in marked regression of the ulcers. PSL tapering induced subcutaneous nodularity with ulceration, and thus, clarithromycin treatment was kept to date. After chemotherapy for 15 months, epithelialization was almost achieved. Valganciclovir was administered when CMV viremia reappeared.

Metformin (an oral antidiabetic) and sitagliptin phosphate hydrate (a selective inhibitor of dipeptidyl peptidase-4) continued for controlling type 2 diabetes mellitus. Mitiglinide calcium hydrate (a stimulator of insulin secretion) was added after the appearance of the skin ulceration for one year. During the clinical course, the patient's blood glucose levels were persistently hyperglycemic, ranging from 106 to 186 mg/dL but without glycosuria. Mild proteinuria ranging from 15 to 100 mg/dL was noted, indicating the complication of stage 2 (early) diabetic nephropathy. His HbA1c levels ranged from 6.4 to 7.3% (standard levels: 4.1–6.2%). The patient thus suffered from mild and controlled diabetes mellitus.

**Figure 6:**
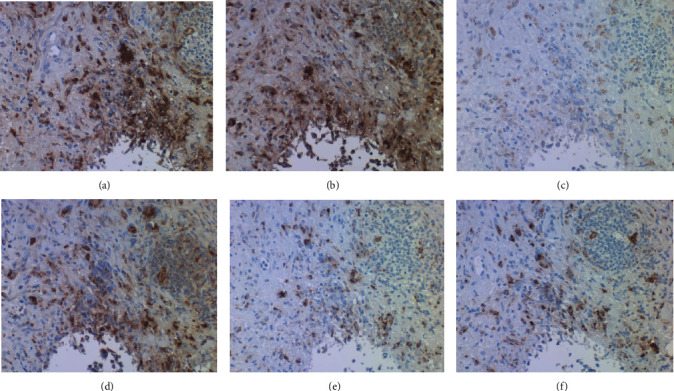
Immunohistochemical demonstration of varied bacterial antigens ((a) BCG, (b) *Bacillus cereus*, (c) *Treponema pallidum*, (d) MPT64, (e) LAM, and (f) PAB). The cytoplasm of macrophages infiltrating around the fat droplet exhibits clear cross-reactivity with varied bacterial antigens. Immunoreactivity of *T. pallidum* antigens is relatively weak.

For characterizing the mycobacteria colonizing the skin lesion in the second and third skin biopsy specimens, immunostaining using selected antibodies was performed, as indicated in Table 1. An amino acid polymer method (Simple Stain Max-PO, Nichirei Biosciences, Tokyo) was employed. As reported in the previous article describing a low-specificity and high-sensitivity immunostaining with widely cross-reactive antibacterial antisera [5], the mycobacteria were visualized in formalin-fixed, paraffin-embedded sections. The antibodies gave clearly positive signals in the cytoplasm of macrophages infiltrating in the lesion, in addition to long rods labeled with Gram and Ziehl-Neelsen stains.  Figure 6 illustrates positive findings with rabbit antisera to Bacillus Calmette-Guérin (BCG) [6], *Bacillus cereus* [5], MPT64 (also called as protein Rv1980c, *M. tuberculosis*-specific 24 kDa secretory antigen) [7], and monoclonal antibodies to LAM (*M. tuberculosis*-related lipoarabinomannan) [8] and PAB (*Propionibacterium acnes*-specific lipoteichoic acid) [9]. Antiserum to *Treponema pallidum* gave weak immunoreactivity. *Escherichia coli* antigens were scarcely observed. The background staining was minimal.

## 3. Discussion

Opportunistic infection caused by a variety of bacteria, fungi, or viruses occurs in the skin under the immunosuppressive state such as acquired immunodeficiency syndrome (AIDS) and after the use of immunosuppressive agents [1, 2]. One of the authors (YT) described histopathological features of such conditions in a textbook entitled *Pathology of Skin Infections* published in 2013 [10]. We describe herein a case of cutaneous coinfection of CMV and *M. chelonae*, provoked by immunosuppressive therapy against pyoderma gangrenosum. The patient suffered from mild and controlled type 2 diabetes mellitus. Pyoderma gangrenosum is characterized by noninfectious autoimmune-type progressive skin ulceration [11], successfully treated with immunosuppressive agents [12].

Opportunistic skin infection of CMV is rare. CMV mainly affects vascular endothelial cells and perivascular stromal cells, and multifocal anogenital ulcerations are most frequently encountered in AIDS patients [13]. Cutaneous lesions with verrucous elevations [14] and septal panniculitis [15] have also been described in non-AIDS immunosuppressed patients. Since the skin ulcer remained unchanged after ganciclovir treatment, Grushchak et al. regarded CMV infection as a bystander phenomenon [16]. The skin lesion in the present case demonstrated features of septolobular panniculitis with CMV inclusions in endothelial and perivascular stromal cells, but the lesion progressed to form large induration with multiple ulcers after gancyclovir treatment. It is reasonable to consider the CMV infection as bystander incidence associated with nontuberculous mycobacteriosis. In other words, when CMV inclusions are noted in nodular or ulcerated skin lesions, primary causative microbes other than CMV should be searched under the microscope, as was so in the present case.

Varied nontuberculous mycobacteria affect the skin, including *M. avium-intracellulare* complex, *M. marinum*, *M. ulcerans*, *M. fortuitum*, and *M. chelonae-abscessus* complex [17, 18]. Reportedly, rapidly growing mycobacteria (RGM) form colonies on a common chocolate agar medium when the culture period is prolonged to two weeks or more [19].

*M. chelonae* was first isolated from the tortoise by Friedmann in 1902 [20]. *M. chelonae* belongs to nonchromogenic RGM (Runyon group IV), widely distributed in environmental water [21]. This zoonotic mycobacterium, categorized in *M. chelonae-abscessus* complex, causes opportunistic infection in the human skin, as well as infection in aquatic animals such as the reptiles and fish [22]. Of notice is that not only traumatic injuries but also surgical procedures make risk factors of human infection [23]. It is plausible that in the present case, medical disposals caused iatrogenic infection of the environmental pathogen. Immunosuppression and diabetic conditions accelerated opportunistic infection, as reported previously [17, 18, 22]. Microscopically, infection of RGM, including *M. chelonae*, provokes abscess-forming granulomas (suppurative granulomas): some lesions are recognized as abscess surrounded by abortive granuloma formation, while the others predominantly reveal epithelioid granulomas [24, 25]. Acid-fast bacilli are characteristically identified in the lipid droplets (vacuoles) often located in the center of suppurative granulomas. It is noteworthy that the bacilli are positively labeled with Gram stain: namely, Gram positivity is a feature of RGM [26].

The cell wall of mycobacteria contains voluminous mycolic acids, extremely long fatty acids, as molecular forms of mycolic acid-containing glycolipid such as trehalose dimycolate and trehalose monomycolate, giving lipophilic nature of mycobacteria [27]. RGM are characterized by the presence of an additional mycolate, glucose mycolate, on the cell wall [28]. Such biological features may be related to the lipid droplet-centered pattern of infection by RGM.

Lipid droplet-centered suppurative granulomas are commonly observed in granulomatous mastitis caused by a lipophilic bacterium, *Corynebacterium kroppenstedtii* [29]. The Gram-positive rods are uniquely clustered in the lipid droplets surrounded by suppurative granulomas. The histological features are quite similar to those of RGM infection. Unlike other corynebacteria, *C. kroppenstedtii* lacks mycolic acid but instead contains tuberculostearic acid in the cell wall [30]. It is understandable from both the microscopic appearance and the pharmacodynamic properties of antibiotics [31] that administration of lipophilic antibiotics is essential for treating not only granulomatous mastitis but also RGM infection. The treatment regimen for tuberculosis using hydrophilic antibiotics such as isoniazid, ethambuthol, pyrazinamide, and streptomycin is consistently ineffective for infection of RGM, which are usually resistant to lipophilic rifampicin [18]. In the present case, clarithromycin, a type of lipophilic macrolides, was effective, as has been reported so far [32, 33]. Thermotherapy was also useful [34], based on the biological features of the bacteria showing low optimal temperature (25–37°C) for growth [17, 18].

Immunohistochemical cross-reactivity of rabbit antisera against BCG and *B. cereus* was observed in the infected lesion. *T. pallidum* antiserum also showed weak cross-reactivity. Positive signals with low background staining were observed not only on the mycobacteria in the lipid droplets but also in the cytoplasm of macrophages, so that the detection sensitivity was very high [5]. Similar results were observed in granulomatous mastitis [29]. The visualization of microbes within the lesion is essentially important for making a histopathological diagnosis of infection [5]. Another important point of our findings includes the cross-reactivity of antibodies against MPT64, LAM, and PAB to *M. chelonae*. It has been reported that MPT64 and LAM are specific to *M. tuberculosis*, and PAB is solely expressed on *Propionibacterium (Cutibacterium) acnes* [7–9]. The *P. acnes* antigens were detected in granulomas of sarcoidosis [35]. The lipophilic bacteria in granulomatous mastitis also expressed LAM and PAB (Tsutsumi, unpublished observation). Such unexpected cross-reactivity should be cautious about applying the antibacterial antibodies as immunohistochemical probes, as has been described previously [5].

## 4. Conclusion

We reported here rare opportunistic skin coinfection of CMV and *M. chelonae*, a rapidly growing nontuberculous mycobacterium, after immunosuppressive therapy against pyoderma gangrenosum. It is of note that *M. chelonae* provoked suppurative granulomas with lipid droplet-centered colonization of Gram-positive and acid-fast rods. Unique immunohistochemical reactivities with a variety of antibacterial antibodies were applicable to visualizing the causative mycobacteria in the lesion.

## Figures and Tables

**Table 1:** Antibodies used in the present study.

Target antigen	Animal/clone	Dilution	Antigen retrieval	Source
Bacillus Calmette-Guérin (BCG)	Rabbit antiserum	1 : 10,000	None	Agilent
MPT64 (RV1980C, 24 kDa protein)	Rabbit antiserum	1 : 800	HIER in 10 mM CB (pH 6)	Abcam
LAM (lipoarabinomannan)	Mouse TMDU3	1 : 1,000	HIER in 10 mM CB (pH 6)	MBL
PAB (Propionibacterium acnes-specific lipoteichoic acid)	Mouse TMDU2	1 : 4,000	HIER in 10 mM CB (pH 6)	MBL
Bacillus cereus	Rabbit antiserum	1 : 500	HIER in 10 mM CB (pH 6)	Abcam
Treponema pallidum	Rabbit antiserum	1 : 1,000	HIER in 1 mM EDTA (pH 8)	BM
Escherichia coli	Rabbit antiserum	1 : 20,000	Proteinase K digestion	Agilent

MPT: mycobacterial protein tuberculosis; HIER: heat-induced epitope retrieval; CB: citrate buffer; EDTA: ethylenediamine tetraacetic acid; Agilent: Agilent Technologies (Santa Clara, CA, USA); MBL: Medical and Biological Laboratories (Nagoya, Japan); Abcam: Abcam plc (Cambridge, UK); BM: Biocare Medical LLC (Pacheco, Philippines).
